# An Iterative Framework for EEG-based Image Search: Robust Retrieval with Weak Classifiers

**DOI:** 10.1371/journal.pone.0072018

**Published:** 2013-08-20

**Authors:** Marija Ušćumlić, Ricardo Chavarriaga, José del R. Millán

**Affiliations:** École Polytechnique Fédérale de Lausanne, Lausanne, Switzerland; University of Bath, United Kingdom

## Abstract

We revisit the framework for brain-coupled image search, where the Electroencephalography (EEG) channel under rapid serial visual presentation protocol is used to detect user preferences. Extending previous works on the synergy between content-based image labeling and EEG-based brain-computer interface (BCI), we propose a different perspective on iterative coupling. Previously, the iterations were used to improve the set of EEG-based image labels before propagating them to the unseen images for the final retrieval. In our approach we accumulate the evidence of the true labels for each image in the database through iterations. This is done by propagating the EEG-based labels of the presented images at each iteration to the rest of images in the database. Our results demonstrate a continuous improvement of the labeling performance across iterations despite the moderate EEG-based labeling (*AUC* <75%). The overall analysis is done in terms of the single-trial EEG decoding performance and the image database reorganization quality. Furthermore, we discuss the EEG-based labeling performance with respect to a search task given the same image database.

## Introduction

Successful decoding of brain signals during rapid serial visual presentation (RSVP) triggered the idea of using the electroencephalography (EEG) signals as an extra information channel for image retrieval. By exploiting the neural correlates of visual recognition of target images in combination with computer vision techniques, both the robustness and flexibility of the human visual system are retained, as well as the speed of computer vision (CV) techniques when dealing with large image collections. Furthermore, CV techniques encounter a problem known as a semantic gap. It represents the difference between a computational representation of the image and the semantic descriptions that users might employ in any given context. Hence, the rationale for EEG-based image search is to link information decoded from brain activity to the semantic description of the presented images. Thus, users can be engaged in the retrieval process by guiding computer vision directly through their EEG channel. In this paper we revisit the framework for brain-coupled image retrieval that relies on the closed-loop synergy between EEG-based image labeling and content-based image retrieval [1].

Previous studies demonstrated that the EEG signature of visual recognition under RSVP protocol can be successfully detected on single trials and applied for real-time image triaging [2], [3]. A combination of BCI based image triaging [2] and CV techniques was later tested using single-object color images from the Caltech dataset [4]. The EEG based labels of the presented images were propagated to a larger image database based on image similarity.

This approach was extended to a closed-loop system [1] where the system may query the user for more information, by means of a new RSVP sequence. At each iteration, a set of the EEG-based labels was evaluated and if it did not satisfy a pre-determined criterion, then a new image sequence was presented. The EEG-based labels were refined with each additional RSVP sequence. Once the criterion was fulfilled, images in the database were ranked by propagating the final EEG-based labels. For most of the subjects the stopping criterion was reached after the second iteration. Reported results show that performance was highly dependent on the type of target image, in particular due to task-dependent variations of the CV performance.

Image retrieval applications deal with a large pool of images to be searched through, in order to find a small portion of the images the user is interested in. Thus, a random image presentation under the RSVP protocol is analogous to the visual two-stimuli oddball experiment (i.e., targets interspersed between frequent non-target visual stimuli). Numerous studies have demonstrated that the appearance of the rare stimuli is followed by a cognitive event-related potential named P300 [5]. It has been shown that the amplitude and the latency of the P300 are influenced by the target discriminability and the target-to-target interval in the sequence [6]–[8]. In realistic image databases, however, the images could be semantically and/or visually related, implying possible similarities between target and some non-relevant images, potentially increasing the variability of the EEG responses.

Furthermore, some of the non-target images might be so salient and unique that they may also induce a P300 waveform. Thus, a parallel between the EEG-based image search and the three-stimuli oddball paradigm (i.e., three types of stimuli: rare target, frequent and rare distractors) seems to be more appropriate. In this paradigm two components of P300 have been identified [6], [9]. The earlier component P3a, localized in the fronto-central region of the scalp, is driven by stimulus novelty in the sequence (i.e., the appearance of an uncommon non-target stimuli can also evoke it). The later component P3b, localized in the centro-parietal region, reflects cognitive processes of task-relevant stimuli recognition. Therefore, in the context of image search under RSVP, the P3b component seems to be the most relevant.

Extending previous works [1], [10] on the synergy between content-based image retrieval and the EEG-based BCI, we give a different perspective on iterative coupling between the EEG decoding and the automatic image labeling – i.e., the process of propagating the decoded EEG labels through the image database using CV techniques. In our framework, we trace the labels that are obtained by propagating the EEG labels after every presented RSVP sequence (i.e., iteration). This way, all images in the database are assigned with several multimodal labels (i.e., EEG-based labels and CV-based labels across iterations) and the final ranking is done according to their average values. The requirement of the previously reported approach for the EEG-based image retrieval is to obtain EEG labels accurate enough for the CV part. However, this requirement might be hard to fulfill in the case of natural images. For this reason, our approach does not rely on such a requirement and still yields a continuous improvement of the labeling performance across iterations even in the case of the moderate performance of direct coupling (i.e., CV propagation of the EEG labels). We evaluate the decoding performance of the framework using natural color images of various types (i.e., images of objects in their natural environment), as might be the case in a real-world application.

The paper is organized in the following way. First, we introduce the experimental setup and the protocol used in the study. Afterwards, we present in detail each part of the framework: the EEG-based image labeling and the content-based label propagation, together with the approach for their iterative coupling. Then, the performance of the individual parts as well as the iterative closed-loop synergy is reported and discussed. We close the paper with the concluding remarks.

## Methods

**Figure 1:**
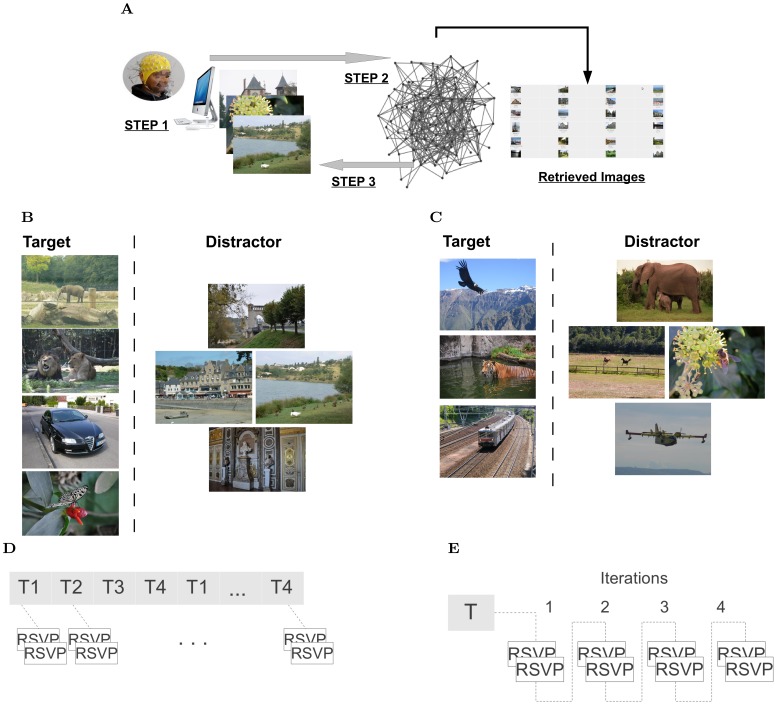
EEG-based image retrieval. (A) The framework: *step 1 -* The RSVP sequence of images, presented to the subject, is labeled based on the recorded EEG signals. *step 2 -* These labels are propagated to the unseen images in the database using CV similarity. *step 3 -* A new RSVP sequence is built from the images top-ranked as target, in accordance to the label propagation results. These steps are repeated iteratively allowing the accumulation of multimodal labels for the images in the database. (B) Illustration of the training images (four different search tasks). (C) Illustration of the testing images (three different search tasks). The images in the figure are similar but not identical to the ones used in the study. Reprinted from http://www.freephotobank.org/main.php and http://animalphotos.info/a/(26-4-2013) under a CC-BY or CC-BY-SA license. Image Copyright is held by original owners. (D–E) The organization of the RSVP sequences in the training and testing phases respectively (T – a search task).

We have designed a framework for EEG-based image retrieval, similar to the one proposed in [1], characterized by the following course of action (Figure 1A). First, an initial RSVP sequence of images is presented to the subject, and images are labeled as target or distractor based on the recorded EEG signals. Second, these labels are propagated to the unseen images in the database based on some CV image similarity measure. Third, a new RSVP sequence is built from the top-ranked images as target, in accordance to the label propagation results. Following a closed-loop setting, these steps are repeated iteratively in order to accumulate evidence of the image labels. A constant number of iterations (N = 4) is used in the study.

We first performed the experimental evaluation of the proposed framework. In addition, we conducted a behavioral experiment measuring the subject’s response time to the target stimuli. Both experiments were run on a subset of the Corel image database that we manually selected. The chosen images represent either images of objects in their natural environment or natural clutter scenes.

### Experimental Setup and Protocol

The study was approved by the Ethics Committee of Canton de Vaud. Fifteen subjects participated in the study. Participants provided their written informed consent to participate in the experiment. All subjects had normal or corrected-to-normal vision. There was no specific criteria for recruiting the subjects. Apart from subjects 1, 2 and 6, they have not performed these type of experiments before. Two of them (Subjects 1 and 2) took part in live demos held during the European Future Technologies Conference and Exhibition (FET11, 4–6th May 2011, Budapest). These demos were given in a public space with high environmental noise. The remaining thirteen subjects were recorded in our laboratory.

#### Data recording

EEG data were recorded with a 64-channel BioSemi ActiveTwo system, in an extended 10–20 montage, at a sampling frequency of 2048 Hz. The peripheral electrodes were not considered to reduce any possible artifacts/noise. The EEG signals, downsampled to 128 Hz, were preprocessed by a fourth-order Butterworth bandpass filter in the range 1 Hz and 10 Hz, since delta and theta activities are known to be related to P300 [11], and downsampled to 32 Hz. Signals were re-referenced based on the Common Average Reference (CAR). On ten subjects (Subjects 6–15) we also recorded the electrooculographic (EOG) activity using three electrodes positioned above the nasion and below the outer canthi of the eyes. Two minutes of calibration data (voluntary eye movements and blinks) were recorded before the experiment started. We used these data to estimate the correction coefficients concerning EOG artifacts [12].

#### Task and stimuli

Subjects were instructed to silently count images of a specified object while natural images were presented to them at a rate of 4 Hz. Although this dual-task can affect the P300 amplitude, it helps in keeping the subject engaged in the task of recognizing target images [6]. Subjects sat at about 60 cm from the screen and the presented images occupy approx 6° × 4° of their visual field.

The training dataset contained diverse images of natural scenes, as well as sample images of four objects (i.e., “Elephant”, “Car”, “Lion” and “Butterfly”, 20 images per object). The testing dataset consisted of natural images organized in 10 different categories (“Aviation”, “Car”, “Dog”, “Eagle”, “Tiger”, “Elephant”, “Wave”, “Horse-jockey”, “Flowers” and “Train”). Figure 1B–C illustrates the type of these images. Note that different sets of images were used in the training (800 different images, presented twice) and testing phases (1382 images).

#### Protocol

The experiment consisted of two phases: training and closed-loop testing. The RSVP sequences were composed of 100 images. In the *training phase* the RSVP sequences were created to satisfy the criteria of an oddball paradigm (10% of the images correspond to the target). There were four different search tasks (i.e., “Elephant”, “Car”, “Lion” and “Butterfly”). Four RSVP sequences were presented per task. In the *testing phase*, the closed-loop was evaluated through four iterations per search task. Two RSVP sequences were presented per iteration. In the initial iteration, 10% of the images were the targets. The content of the RSVP sequences in a given iteration was selected based on the propagation of the EEG-based labels obtained in the previous iteration. All the subjects performed three different search tasks (i.e., “Eagles”, “Tiger” and “Train”). Notice that the target tasks in the *testing phase* were different from the *training phase*. Thus, some images used as target during training might appeared as distractors in the testing phase.

#### Behavioral experiment

The response time (RT) to target images in the RSVP sequence reflects the visual processing behind target discrimination. In the case of natural images, the semantic similarity between the target and distractor images affects the response time to targets [13]. Thus, as a complement to the EEG study, we analyzed target discriminability across the search tasks by means of RT analysis. In the behavioral experiment subjects had to press a key as quickly and accurately as possible whenever there was a target. The same stimuli were used as for the framework evaluation. Ten out of the fifteen subjects took part in the behavioral test. At least two days were left in-between the two experiments to minimize any learning effect. The EEG experiment always preceded the behavioral test. Finally, we examined if the median response time significantly differ across search tasks by means of the Friedman statistical test (a non-parametric test for testing the difference between several related samples).

### EEG-based Image Labeling

The images presented to the user are first labeled by means of the EEG decoding, indicating whether they are interesting for the user in the given context (i.e., target images). To do so, we perform EEG single trial classification in the time domain. The data from the training phase are used to build a classifier. The feature vector is obtained by concatenating samples in the interval from 200 ms to 700 ms after stimulus onset of a subset of 8 channels: C3, Cz, C4, CPz, Pz, PO3, POz, PO4. These channels are chosen based on the centro-parietal scalp distribution of P3b subcomponent [6], [9] and previous work on channels selection for P300 detection [14]. Since 128 features (time samples by channels) are too many to build a robust classifier with a limited number of samples, we select a subset by computing their discriminant power (DP) separately on three folds of the training data. The discriminant power of features is evaluated using a Canonical Variate Analysis (CVA) based method [15]. For a two-class classification problem, it scores a feature based on its correlation with the data projection onto the decision vector. Finally, the most discriminant features (DP>1%) across all folds are kept, so as to select stable discriminant features. The average number of the selected features (time samples by channels) across subjects is 21.6 ± 4.7. Target vs. distractor trials classification is performed by a Gaussian classifier [16], using four prototypes per class. A Gaussian classifier is a generalization of well-known LDA and QDA [17], where each class may be represented by several clusters (or prototypes). The output of the classifier is an estimation of the posterior class probability distribution.

#### EOG artifacts

To eliminate the EOG as a potential source of the discriminant activity we did an offline evaluation of the single trial classification using the EOG corrected EEG signals. For this purpose we apply an automated correction method based on regression analysis [12]. Assuming the independence between the uncorrupted EEG and EOG signals, the correction coefficients were estimated on the data recorded in the calibration session (see *Experimental Setup*). Topographical representation of the estimated coefficients averaged across the subjects is given in Figure S1.

### Image Label Propagation

Once the images presented under RSVP are labeled based on the recorded EEG, the remaining images in the database are labeled by means of a propagation technique. For this we use a semi-supervised approach derived in a Bayesian Network framework, based on a visual similarity graph of the images in the database [18].

#### Graph

Each node in the graph represents one image. The state of a node is the probability that the image belongs to a certain class (target or distractor); i.e., it corresponds to the label of the image. In turn, an arc between two nodes represents the conditional probability that these nodes belong to the same class, taking into account their similarity in the CV feature space. The images in our database are indexed in two CV feature spaces: (*i*) the colored pattern appearance model (CPAM) [19], characterizing image patches in terms of the chromatic and achromatic spatial patterns, and (*ii*) the edge histograms [20]. First, we build a similarity graph for each feature space where every node is connected to K nearest EEG-based labeled and to the same number of the nearest unlabeled nodes. We consider (K = 5), as no improvement is observed for higher numbers. Prior to label propagation, we merge the graphs into a common graph (joint model), giving them equal contribution. This is done under the Bayesian framework as the states of nodes and arcs between them represent probabilities.

#### Label propagation

EEG-based labels are propagated to unseen images by solving a quadratic optimization problem on the graph [18]. The objective is to minimize the difference between the current image labels and the labels estimated based on the neighboring nodes, while the available EEG-based labels are taken as a constraint. Note that the EEG-based labels are hardened prior to the propagation (i.e., values 1 and 0 correspond to classes target and distractor, respectively) and they are not changed in the propagation process. However, the propagation over the unlabeled nodes results in soft CV-based labels (i.e., labels are continuous values in the range [0–1]).

#### Label balancing

To avoid biased label propagation towards one of the classes, prior to the propagation we undersample the EEG-based labels in order to have the same number of examples of targets and distractors. This is done by random elimination of labeled images from the class with the majority of examples.

### BCI Image Search: Iterative Approach

After the propagation of EEG-based labels to the rest of images in the database, a new RSVP sequence for the next iteration is generated with the top 200 images ranked as targets. By repeating these steps we are accumulating evidence of the true labels for each image in the database. Four iterations are considered in the analysis. After the final iteration, the database is reorganized based on the averaged labels (i.e., the states of the graph) across iterations. Nevertheless, iterations are run independently; i.e., only the EEG-based labels obtained in the current iteration are used to select the images for the next iteration. The retrieved images are the top 200 ranked images in accordance with the final image labels.

As mentioned before, the applied label propagation does not update the EEG labels. Hence, due to the binary EEG-based labeling (target vs distractor) of the presented images, images recognized as target in the current iteration will be included in the next RSVP sequence. Nevertheless, since the final image labeling is obtained by averaging the accumulated labels (labels after propagation) over all iterations, the approach indirectly update the labels of the presented images.

Previous work on iterative coupling [1] improves the set of the EEG-based labels across all iterations (average labels are used for images presented multiple times) before propagating them to the unseen images for the final labeling of the database. To test this approach in our setting, we did an offline evaluation of image retrieval and compared it with the method that we proposed.

## Results

### EEG-based Image Labeling

**Figure 2:**
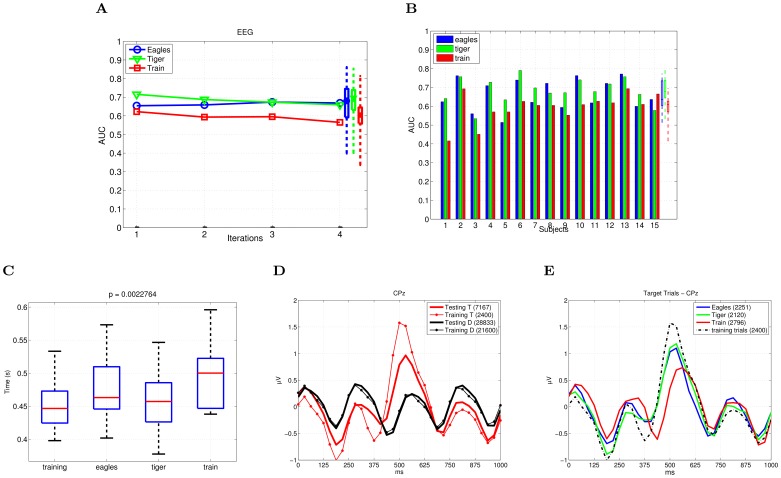
EEG-based Image Labeling. (A) Average EEG-based labeling performance (AUC) across subjects. (B) Single trial EEG classification performance (AUC) on the EEG data averaged across the iterations, given for each subject individually. The rightmost boxplots show the median (the central mark of the box), the 25th and 75th percentiles (the edges of the box) and the most extreme performance (dashed lines) over all subjects per search task. (C) Median response time (in seconds) across subjects for the different search tasks. (D–E) Grand Average of the potentials evoked by the target and distractor images at CPz: Training vs. testing sequences and Individual testing tasks as compared to the training task.

We report the single-trial EEG classification performance per iteration, averaged across subjects for each search task in the testing phase: “Eagles”, “Tiger” and “Train”, (Figure 2A). The performance is given in terms of the area under the curve (AUC) of a receiver operating characteristic (ROC) curve [21]. For each search task, a Wilcoxon signed-rank test shows a statistically significant difference between the EEG-based labeling performance across subjects and the random labeling of the same RSVP sequences (p< 0.05 ). Random labels are drawn (100 repetitions) from the uniform distribution in the interval (0,1) to simulate the random labeling.

The average performance across iterations is given separately for each subject in Figure 2B. The decoding performance in the noisy environment (Subjects 1 and 2) is comparable to the one obtained with the recordings performed in the laboratory. No significant difference is found on the average performance after applying the EOG-correction (*p*>0.05, Wilcoxon sign-rank test). The supplementary material (Text S1) provides additional results of the EOG analysis and illustrates the averaged EOG waveforms and the averaged EEG responses after the correction of EOG artifacts, Figure S2.

As pointed out earlier, image search under RSVP protocol is considered as an oddball paradigm, therefore depending on the relative appearance of the target stimuli (i.e., target-to-target interval TTI). In the testing phase of our experiment, the TTI may vary greatly, as a consequence of the automatic generation of the RSVP sequences and the prevalence of targets. Thus, we evaluate the EEG classification performance distinguishing four categories of target trials with respect to TTI (i.e., TTI = 1, 2, 3, >3), where the TTI is given as the distance of a target image to its preceding target in the RSVP sequence. A significant drop in the performance (*p*<0.05, Wilcoxon signed-rank test) is found for the successive targets (TTI = 1) compared to the other three conditions in all of the search tasks, except (TTI = 3) condition in the search task “Train”. Furthermore, the difference between (TTI>3) and (TTI = 3, “Eagles’)/(TTI = 2, “Tiger”) is statistically significant.

On the other side, we compare the classification performance between the search tasks. Overall, the lowest performance is obtained for the search task “Train”. A Wilcoxon signed-rank test shows a significant difference (p<0.05) in the classification performance between the search task “Train” and the other two tasks (TTI = 3, >3).

We hypothesize that target discriminability varies across the tasks, causing the task dependent performance. The high performance for class “Tiger” can be explained by its salience in the sequence (the characteristic pattern of tiger’s stripes). On the other side, the search task “Train” is characterized with a higher intraclass variability (e.g., different colors and models of train). Furthermore, the similarity with the images of objects such as “Car” and “Aviation”, which appeared among the distractor images, made the task “Train” more challenging. The results of the behavioral experiment support our hypothesis. There is no significant difference (Friedman, p>0.05) in the median response time between the different targets in the training phase. However, there is a significant difference (p<0.05) between the targets in the testing phase, as well as between the training targets and the target “Train” in the testing phase (Figure 2C). The longest median response time is observed for the target “Train”, indicating its lowest discriminability.

Furthermore, from the results of the behavioral experiment we observed that the subjects’ responses are more accurate in the training sequences. On average, the subjects responded correctly to 98.9% of the training targets (98.8% “Elephant”, 99.5% “Lion”, 98% “Car”, 99.3% “Butterfly”) and to 90.63% of the testing targets (90% “Eagles”, 94.8% “Tiger” and 90.1% “Train”). Figure 2D–E shows the grand average of the potentials evoked at CPz by the different search tasks. Note the lower amplitude of the peaks associated to the testing search tasks, with the target “Train” eliciting the smallest peaks. For this later target, peaks are also delayed with respect to all others.

### EEG-based Image Search: Iterative Approach

We assess the image search performance using the average precision (AP), since the system returns a ranked sequence of images. The precision score of a target image in the retrieved sequence is computed as *i*/*n*, where *i* is its position among the targets in the sequence and *n* is its absolute position in the sequence. If one target image is not in the retrieved sequence its precision score is zero. Then, AP is defined as the mean of the precision scores over all target images [22].

**Figure 3:**
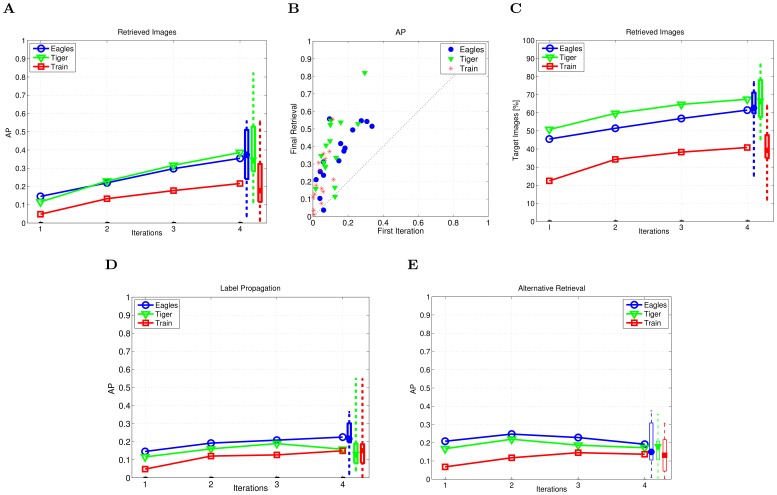
Closed-loop retrieval performance evaluated on testing tasks (i.e., “Eagles”, “Tiger” and “Train”). (A) Average precision by successive averaging. (B) Average precision of the 200 retrieved images after the first iteration vs the final retrieval (closed-loop with successive averaging). (C) Retrieved target images after each iteration in respect to the total number of targets in the database. (D) Average precision of the 200 top ranked images obtained by propagating only the harden EEG-based labels obtained for the last seen sequence; (E) Average precision of the 200 retrieved images using the approach for the iterative EEG-based image retrieval in [1]. Note that, after propagation of EEG-based labels, images in the database that were included in the presented RSVP sequences have the original soft EEG-based labels for this approach, while our approach assigns them hard labels. The rightmost side of the plots show the performance across subjects in the last iteration. The median (the central mark of the box), the 25th and 75th percentiles (the edges of the box) and the most extreme performance (dashed lines) across subjects, are reported per search task for the final database ranking.

One can notice persistent improvement across iterations in the AP of the retrieved 200 images (Figure 3A). On average, the AP after four iterations reaches 0.37 (class “Eagles”), 0.39 (class “Tiger”) and 0.22 (class “Train”). Figure 3B shows, for all subjects, that the AP for the final retrieval (y-axis) is consistently higher than for the initial iteration (x-axis).

Furthermore, the percentage of retrieved target images also increases due to the iterative coupling (Figure 3C). In the initial RSVP sequence 10% of images are targets, which corresponds to 22% (class “Eagles”), 27% (class “Tiger”) and 10% (class “Train”) of all target images in the database. After the final ranking, on average, the percentage of the retrieved target images is 62% (class “Eagles”), 68% (class “Tiger”) and 40% (class “Train”).

The strength of the system is not in one of the components but in the iterative coupling. The performance of simply propagating the latest EEG labels is given in Figure 3D. No increase in the AP is noticed after the second iteration. However, the results are significantly different from the propagation of the random labels that are assigned to the presented images (p<0.05, Wilcoxon signed-rank test). Moreover, when applying a propagation where the EEG labels obtained in the previous iterations are averaged before being propagated, as in [1], there is no increase in the AP after the second iterations (Figure 3E).

**Figure 4:**
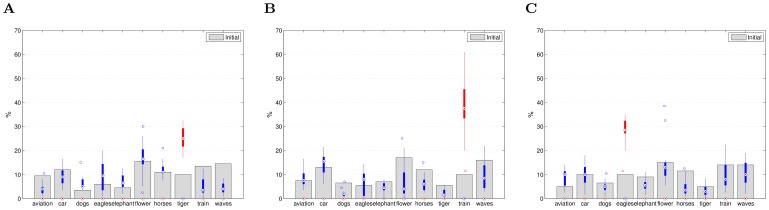
Distributions of the images in the initial RSVP sequences (gray bars) and the retrieved set of images, after the last iteration, over the classes. Distributions of the retrieved images are given across the subjects as a boxplot: the median (the central mark of the box), the 25th and 75th percentiles (the edges of the box) and the most extreme values (thin lines). Red color indicates the target class. (A) Search task: “Tiger”. (B) Search task: “Train”. (C) Search task: “Eagles”.

The image distributions of the initial RSVP sequences over the classes in the database are given individually for each search task in Figure 4A–C. No bias toward the target class can be noticed. In the same figure, distributions of the retrieved images (after the last iteration) are given as a boxplot across the subjects. One can notice that the distributions are largely in favor of the target class, across the subjects and the search tasks.

These results demonstrate that the iterative closed-loop design improves the initial performance (first iteration) despite the relatively high false positive (FP) rate of the EEG-based labels.

## Discussion

The EEG-based image search is founded on the supervised EEG decoding. Thus, knowing that the EEG signature associated to natural images may differ depending on the search task, its performance heavily relies on the training image examples. But selecting a representative set of example images is a daunting task. We have then revisited the framework for brain-coupled image search [1], [2], [10] and analyzed the effect of the target class using natural images.

We have shown that the performance of the EEG single-trial classification is affected by the target image class. We explain it as a result of the changes in the EEG signature caused by variation in target discriminability [6], [7]. The behavioral response to different target images across subjects supported our hypothesis, as the EEG classification performance were the poorest for the target class with the largest median response time across subjects. This points out the existence of behavioral and perceptual target-dependent differences when dealing with natural images. In turn, this variability is likely to be found also in the discriminability of the elicited EEG signals (see Figure 2D–E). This fact should be taken into account so as to build decoding systems that properly reflect the stimulus diversity that may be encountered. It is worth noting that all images come from the same database implying that in real conditions it is difficult to control the image discriminability.

In addition, CV features may not be able to capture all the particularities of a large variety of natural images. Because of the intrinsic limitations of EEG-based labeling and CV features, we didn’t refine the available EEG-based labels through iterations as proposed in [1], [23]. Instead, we exploited the multimodal labels (EEG-based and CV-based), accumulated through iterations, for the final ranking.

The results obtained in our setting by propagating the EEG-based labels averaged across the iterations, as in [1], are inferior to those obtained by successive averaging of the results of label propagation across the iterations (Figure 3A and E). The reason is that the quality of the EEG-based labels, in terms of the false positive rate, does not significantly improve throughout the iterations (*FP*>0.6). On the other side, the successive averaging of the labels obtained after propagation in each iteration can be interpreted as the fusion of the weak classifiers decision, what explains the lower sensitivity to the single-trial misclassification.

**Figure 5:**
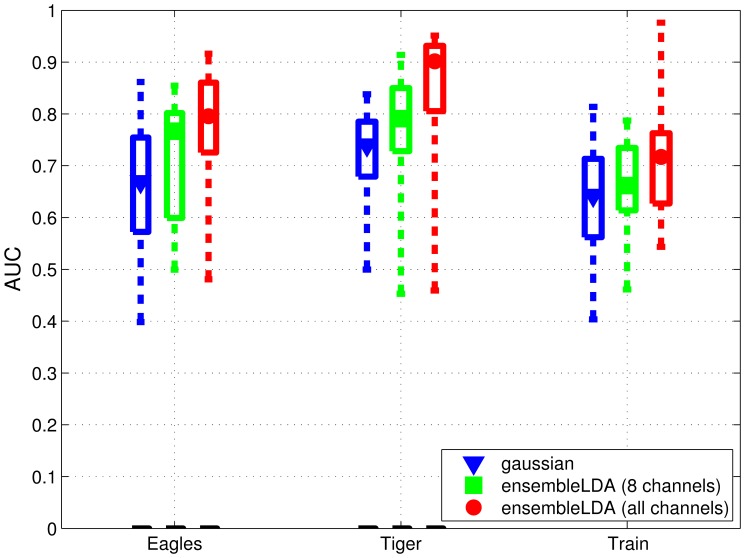
Classification performances comparison (AUC) for different number of channels and classifier types. The data from the initial RSVP sequences in the testing phase are used. The median (the central mark of the box), the 25th and 75th percentiles (the edges of the box) and the most extreme performance (dashed lines) over the subjects are reported per search task.

In this study we have constrained our analysis to centro-parietal electrodes. We compare how performance changes when all channels are taken into account, instead of that small set of channels. Since the increased input dimensionality makes impractical the use of the Gaussian classifier, we used instead an ensemble of LDA classifiers [2]. This method fuses several LDA classifiers (one per each time window of 100 ms) and exploits all the channels (41 channels in our case), but also allows channel reduction. Figure 5 shows the results for the initial RSVP sequence of the testing phase. In both cases, the input is the time window from 200 ms to 700 ms after stimulus onset. We can make three observations. First, the influence of the target class on the classification performance is confirmed. The classification for target class “Train” underperforms the other two classes irrespective of the method. Second, when the analysis is limited to the subset of centro-parietal channels, we observe that the classifier ensemble outperforms the single Gaussian classifier. This may be due to temporal variance in the neural signature, which can be better modeled by the classifier ensemble since each classifier corresponds to a different temporal interval of a trial. Third, the results revealed a lower performance when only the subset of centro-parietal channels is used.

### Conclusion

Keeping in mind practical applications, we demonstrated that a limited number of EEG channels provide sufficient information about subject’s preference to be exploited in image retrieval by the proposed synergistic scenario (e.g., by coupling CV and EEG single trial classification).

Furthermore, as for the observed behavioral responses, the discriminability of the elicited EEG signals exhibit task-dependent variations when dealing with natural images. As a consequence, this effect should be taken into account so as to build decoding systems that properly reflect the stimulus diversity that may be encountered when working with natural images. In this work we have shown how an iterative framework for EEG-based image search can yield a robust retrieval with moderate EEG classifiers.

It is worth noticing that the proposed system was tested outside the laboratory in a real-world environment. Remarkably, the performance obtained in this setting was comparable to the one obtained in the laboratory.

## Supporting Information

Figure S1 **EOG artifacts correction.** Topography of the correction coefficients (*left* central-left, *right* cental-right).
(TIFF)

Figure S2 **EOG and EEG waveforms.** (A–F) Grand average EEG ERPs with and without EOG correction across ten subjects; (G–J) Grand average EOG ERPs: (G–H) Ten subjects, (I–J) Nine subjects. The intervals of significant difference between the two conditions are marked in blue (two-sample t-test, at the 5% significance level).
(TIFF)

Text S1 **Analysis of EOG signals in the EEG-based image search.**
(PDF)
