# Supplementary figures and images for: An Iterative Framework for EEG-based Image Search: Robust Retrieval with Weak Classifiers
Source: PLoS One. 2013 Aug 20;8(8):e72018. doi: 10.1371/journal.pone.0072018 (PMC3748021; doi:10.1371/journal.pone.0072018)

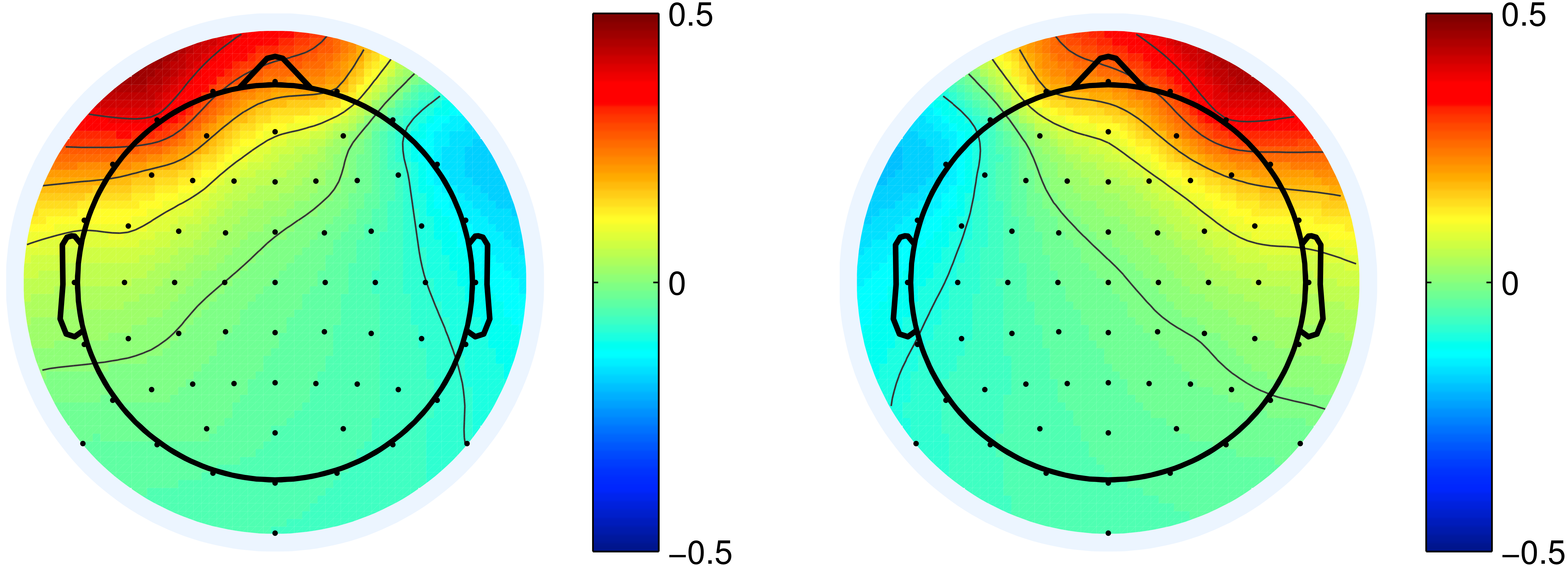

Supplement: Figure S1 — EOG artifacts correction. Topography of the correction coefficients (left central-left, right cental-right). (TIFF) [file pone.0072018.s001.tiff]
