# Supplementary material for: An Iterative Framework for EEG-based Image Search: Robust Retrieval with Weak Classifiers
Source: PLoS One. 2013 Aug 20;8(8):e72018. doi: 10.1371/journal.pone.0072018 (PMC3748021; doi:10.1371/journal.pone.0072018)
Supplement: Text S1 — Analysis of EOG signals in the EEG-based image search. (PDF) [file pone.0072018.s003.pdf]

Text S1:

Analysis of EOG signals in the EEG-based image search

Signals from eye movements can be orders of magnitude larger than brain activity potentials. Therefore, a presence of eye movements introduce significant artifacts in electroencephalogram (EEG) signals.

We monitored eye movements throughout the experiment using three electrooculogram (EOG) electrodes placed above the nasion, and below the outer canthi of the eyes. In the analysis we considered bipolar EOG activity – "central-left" and "central-right".

For the correction of the EOG artifacts in EEG recordings we applied the method proposed in [12]. The correction coefficients estimation in this method requires the calibration data recorded while subjects performed voluntary eye movements and blinks. Thus, at the beginning of the experiment we recorded two minutes of calibration EEG and EOG signals. Topographical representation of the estimated coefficients averaged across the subjects is given in Figure S1. As expected, the influence of eye-movements is dominant in the frontal scalp regions while rapidly diminishes towards the occipital regions.

We looked at the event related EEG potentials (ERPs) corresponding to an image appearance in RSVP sequences. The grand average ERPs at the frontal channels F_z, F_7 and F_8, before and after the EOG artifact correction are given in Figure S2A-F. One can notice that the correction introduce minor changes in the grand average ERPs. The grand average of the event related EOG potentials are given in Figure S2G-J. A low range of amplitude indicates minor eye activity that is locked to the stimuli. However, one can notice a significant difference between the two conditions (target and distractor) which is more evident in the training phase of the experiment. Nonetheless further inspection of the signals showed that by excluding one of the subjects from the analysis the dominant peak (at around 600ms of stimulus onset) in the training phase disappears and no significant difference is found between the two conditions (Figure S2I-J).

Finally, we compared how single trial classification performance changes when only EOG channels are considered, instead of the 41 EEG channels (the peripheral electrodes were not considered). We applied an ensemble of LDA classifiers on the interval from 200 ms to 700 ms of stimulus onset. The average performance across subjects is AUC = 0.57 $\pm$0.07, while the EEG-based classification performance is remarkably higher AUC = 0.75 $\pm$0.1.

We can conclude that the EEG-based target detection reported in this study is not driven by eye movements.
